# Role of Vector Flow Mapping in Evaluating Left Ventricular Diastolic Flow Dynamics in Patients Who Underwent Mitral Valve Repair for Degenerative Mitral Regurgitation

**DOI:** 10.31083/j.rcm2309301

**Published:** 2022-09-05

**Authors:** Ying Wang, Yanan Li, Cunying Cui, Zhenwei Ge, Yuanyuan Liu, Yanbin Hu, Danqing Huang, Chengzeng Wang, Lin Liu

**Affiliations:** ^1^Department of Ultrasound, Fuwai Central China Cardiovascular Hospital, Central China Fuwai Hospital of Zhengzhou University, Henan Provincial People’s Hospital, People’s Hospital of Zhengzhou University, 450000 Zhengzhou, Henan, China; ^2^Department of Cardiac Surgery, Henan Provincial People’s Hospital, People’s Hospital of Zhengzhou University, Fuwai Central China Cardiovascular Hospital, 450000 Zhengzhou, Henan, China; ^3^Department of Ultrasound, The First Affiliated Hospital of Zhengzhou University, 450000 Zhengzhou, Henan, China

**Keywords:** vector flow mapping, energy loss, mitral valve repair

## Abstract

**Background:**

Mitral valve (MV) morphology after MV repair affects postoperative left ventricular (LV) blood flow pattern and long-term cardiac function. Pilot data suggest that LV diastolic vortex flow pattern changes after operation, but specific quantifiers remain unknown. We aimed to explore the role of vector flow mapping (VFM) in LV diastolic vortex flow pattern in patients who underwent MV repair.

**Methods:**

A total of 70 patients with degenerative mitral regurgitation were consecutively enrolled and 30 age- and gender-matched controls were recruited. 50 Patients who underwent MV repair were eventually included in our study. LV average energy loss (EL-AVE) during diastole was measured in the MV repair group by VFM one week before and one month after the operation, and compared with that of controls using one-way analysis of variance. The effect of surgical techniques and the extension of leaflet degeneration on postoperative EL-AVE were analyzed using muti-way analysis of variance, and patients were categorized into a resection subgroup (n = 29) and a non-resection subgroup (n = 21).

**Results:**

The EL-AVE one month after operation in the MV repair group was decreased (*p *< 0.001) compared to that one week before the operation, and was increased (*p *< 0.001) compared to that in controls. Mitral leaflet resection had a statistically significant effect on postoperative EL-AVE. The EL-AVE of the resection subgroup was higher than that of non-resection subgroup (*p *< 0.001).

**Conclusions:**

VFM can be used to evaluate the diastolic blood flow pattern of LV after MV repair, and to observe the changes of LV blood flow pattern caused by different surgical techniques. VFM may be a potential new hemodynamic evaluation method after MV repair.

## 1. Introduction

Mitral valve repair is an operative method for the treatment of degenerative mitral regurgitation. It has advantages over mitral valve replacement in terms of survival rate, valve complications, and valve durability [1, 2, 3, 4], and therefore is the first choice for the treatment of mitral regurgitation recommended by the Guide [5]. Currently, long-term function of the mitral valve and left ventricle (LV) after mitral valve repair is important in the management of patients. The description of cardiac flow patterns after surgical provides an intrinsic qualitative evaluation of therapeutic procedures, which is useful in assessing the potential risk of cardiac abnormalities in cardiac function analysis [6]. However, an effective index is still lacking in assessing cardiac fluid dynamics after mitral valve repair.

Echocardiography is typically used to clinically evaluate the surgery, but is difficult to observe the local and global movement of the myocardium in detail, as well as the changes in hemodynamics in the heart cavity. In most heart valve diseases, the hemodynamics in the heart cavity alter prior to the manifestations of clinical symptoms of cardiac dysfunction.

Vector flow mapping (VFM) is a safe, effective, and non-invasive new ultrasound technology to detect changes in hemodynamics in the heart cavity. It also provides visual observation and quantitative evaluation of the fluid dynamics of the cardiovascular system. At present, VFM technology has been applied to study and analyze energy loss. Studies have found that VFM does not only have value for the evaluation of heart function [7, 8, 9], but also has important clinical value for heart valve diseases, such as valve regurgitation or stenosis [10, 11]. VFM has been applied to evaluate surgical procedures and postoperative hemodynamics [12, 13]. The aim of this study was to apply the novel flow visualization echocardiographic technology VFM for the evaluation of the LV vortex flow patterns and average energy loss (EL-AVE) in patients who underwent mitral valve repair.

## 2. Methods

### 2.1 Study Population

**Fig. 1.**
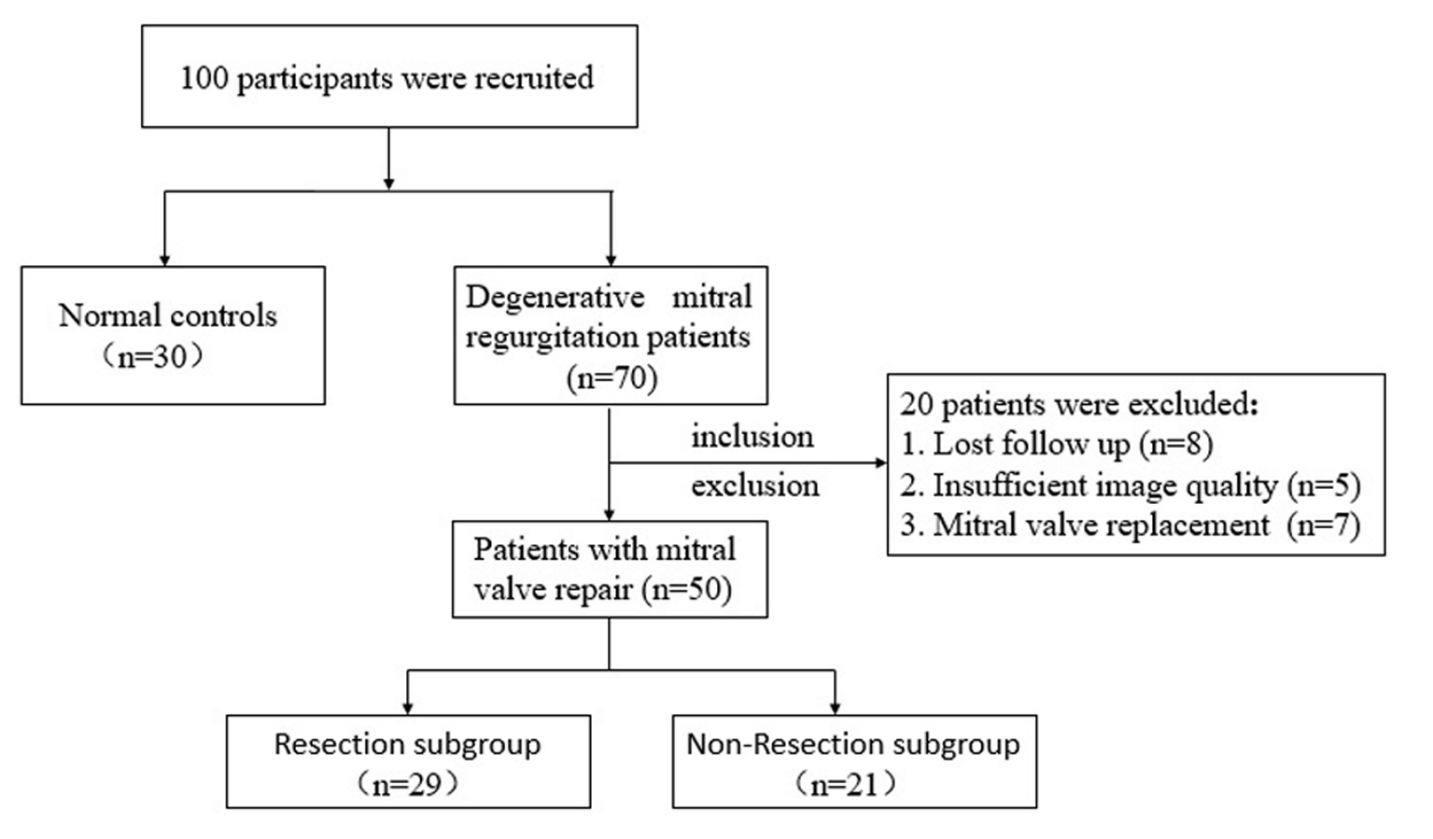
**Study flow chart**.

A retrospective review of VFM in the Mitral Valve Repair program database in our hospital identified patients with a diagnosis of degenerative mitral regurgitation between June 2019 and May 2021. A total of 70 consecutive patients with degenerative mitral regurgitation because of prolapse degeneration of the mitral valve involving single or two leaflet scallops were enrolled. Patients who were lost to follow up, with insufficient quality of images, or with mitral valve replacement were excluded. The final analysis included 50 patients who underwent mitral valve repair by a single surgeon in our hospital. Based on mitral leaflet resection, the patients were divided into two subgroups: 29 patients with mitral leaflet resection (resection subgroup) and 21 patients without mitral leaflet resection (non-resection subgroup) (Fig. 1). All subjects underwent echocardiography, one week before and one month after operation. There was no significant difference in postoperative drug treatment between patients.

To compare patients with degenerative mitral regurgitation with controls of similar age and gender, 30 healthy volunteers were selected as control group during the same period. All included volunteers were confirmed to be free of abnormalities by physical examination, electrocardiogram, X-ray, echocardiography and laboratory tests in a physical examination center. Data on height, blood pressure and weight were collected.

Inclusion criteria were as follows. The subjects were in sinus rhythm, had left ventricular ejection fraction (LVEF) >50%, and the mitral valve regurgitation was caused by degeneration. The degree of mitral regurgitation was based on the American Society of Echocardiography (ASE) diagnostic criteria [14]: moderate and severe mitral regurgitation were defined as an effective regurgitant orifice area (EROA) of 0.30–0.39 ${\mathrm{cm}}^{2}$ and ≥0.40 ${\mathrm{cm}}^{2}$, respectively.

Patients with atrial fibrillation, rheumatic mitral valve, myocardial infarction, cardiomyopathy, other severe valve diseases, hypertension, diabetes, chronic kidney disease, and previous heart surgery history were excluded.

Only patients who underwent mitral valve repair were included. The procedure frequently involves leaflet resection, use of annular rings and neochordae to reshape the annulus and support leaflet repair.

### 2.2 Echocardiography 

The Aloka F75 color Doppler ultrasound system and the UST-52105 heart probe with a frequency of 1–5 MHz were used while the participant was in a left-side lying position, and breathed calmly. An electrocardiogram was simultaneously recorded. Height and weight were assessed to calculate the body surface area (BSA, unit $m^{2}$). Routine echocardiography was conducted; left atrial dimension (LAD) was measured in the long axis view of the LV, and left ventricular end-diastolic dimension (LVEDD), left ventricular end-diastolic volume (LVEDV), left ventricular end-systolic dimension (LVESD), left ventricular end-systolic volume (LVESV), and LVEF were measured using the Simpson biplane method at the apical four-chamber and two-chamber view. Mean transmitral gradient was calculated using the ultrasound system which traces the peak velocity curve at the mitral valve.

### 2.3 Vector Flow Mapping (VFM) 

Dynamic color Doppler blood flow images of the LV chamber were collected from the apical four-chamber view in VFM mode. The probe emission frequency was adjusted to clearly display the endocardium. The maximum velocity range of the color Doppler (Nykist limit) was set at 60–80 cm/s, and the color baseline was kept at 0 cm/s. Under these conditions, the image frame rate was increased as much as possible.

**Fig. 2.**
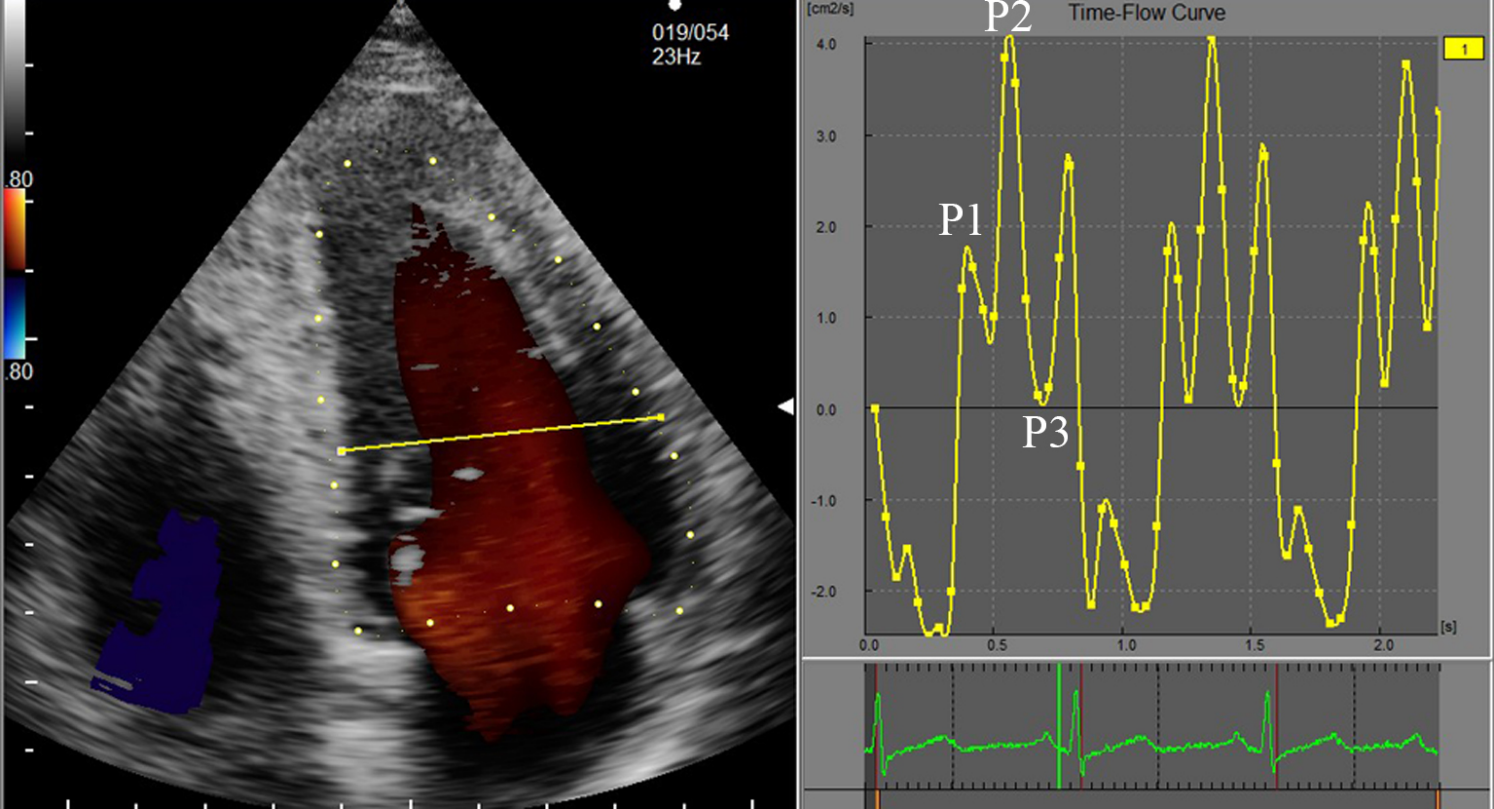
**The LV cardiac cycle time-flow curve**. Each point of the curve corresponds to the frame rate of the ECG. The three periods of the diastole: P1—fast filling period, P2—slow filling period, and P3—atrial systolic period.

**Fig. 3.**
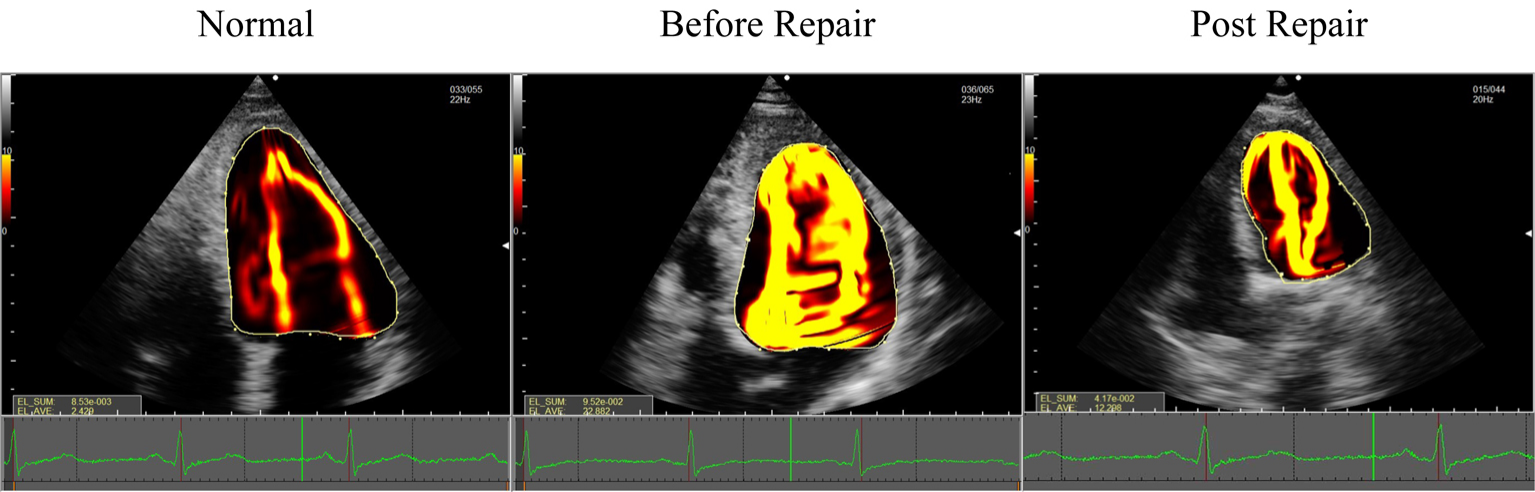
**EL-AVE in patients who underwent mitral valve repair and controls**. EL-AVE of patients after mitral valve repair was higher than that of controls. EL-AVE, average energy loss.

Three cardiac cycles were continuously collected and VFM data was stored on the mobile hard disk for offline analysis. The VFM image data were entered in the DAS-RSI workstation, the analysis interface was entered. The time-flow curve was used to define the time period, and the electrocardiogram (ECG) R-R wave apex was selected as a complete cardiac cycle. Based on the ECG and valve opening and closing conditions, a complete cardiac cycle was divided in three periods: fast filling period (P1), slow filling period (P2), and atrial systolic period (P3) (Fig. 2). The LV diastolic EL-AVE was measured in energy loss mode, and the average EL-AVE value of these three time periods was calculated. The differences in EL-AVE between groups was compared (Fig. 3).

VFM uses blood flow velocity to determine energy loss caused by viscous friction [15]. Intracardiac energy loss is calculated using the following equation:

(1) $$\text{ Energy Loss }=\int \mu \,\left\{2\,{\left(\frac{\partial u}{\partial x}\right)}^{2}+2\,{\left(\frac{\partial v}{\partial y}\right)}^{2}+{\left(\frac{\partial u}{\partial y}+\frac{\delta \,v}{\delta \,x}\right)}^{2}\right\}\,𝑑A,$$

In which μ is the viscosity of the blood, u and v are velocity components on the Cartesian axes (x and y), and A is the area of the unit of the grid.

As seen in the equation, energy loss is the total of squared differences between neighboring velocity vectors. It changes when the size and direction of velocity vector change.

### 2.4 Statistical Analysis

Data were compared using SPSS version 22.0 (SPSS Inc., IBM, Chicago, IL, USA). Continuous variables were presented as mean ± standard deviation or median and interquartile range depending on the distribution. Categorical variables were presented as percentages. Results of two groups were compared using unpaired Student’s *t*-test and chi-squared test. One-way analysis of variance (or Kruskal-Wallis for non-normally distributed continuous variables) was used to examine the difference of variables across >two groups. Statistical significance was accepted at *p *< 0.05.

The effect of postoperative EL-AVE one month after operation was investigated using muti-way analysis of variance, with subgroups of patients with leaflet resection and those without resection, patients with degeneration of mitral valve involving single or two leaflet scallops, and with or without neochordae. The patients in the subgroups were divided based on these factors.

Ten random individuals were selected for evaluation of intraobserver and interobserver agreement on EL-AVE using Bland-Altman analysis.

## 3. Results

### 3.1 Study Participants

We screened 70 patients with degenerative mitral regurgitation and enrolled 50 patients in the study. The most common leaflet abnormality in the patients was prolapse involving the posterior mitral leaflet. The repair procedures included 50 patients with an O-shaped semi-rigid complete ring, 27 patients with neochordae, 29 patients with leaflet resection, and 21 patients with no leaflet resection. There were no statistically significant differences between patients who underwent mitral valve repair and the control group, including age (*p* = 0.64), sex distribution (*p* = 0.78), systolic blood pressure (*p* = 0.41), diastolic blood pressure (*p* = 0.35) and BSA (*p* = 0.38) (Table 1).

**Table 1.** **Clinical characteristics of controls and patients with degenerative mitral regurgitation**.

Variable		Control group	Mitral valve repair group	*p* value
		(n = 30)	(n = 50)	
Age, years		53 (45–61)	56 (47–66)	0.64
Male (%)		18 (60)	29 (58)	0.78
Body Surface Area, $m^{2}$		1.62 ± 0.36	1.60 ± 0.26	0.38
Blood pressure, mm Hg				
Systolic		118.35 ± 4.78	115.3 ± 10.46	0.41
Diastolic		70.46 ± 5.23	68.8 ± 5.74	0.35
Extension of leaflet degeneration				
	Single (%)	—	32 (64)	—
	Two (%)	—	18 (36)	—
Location of leaflet degeneration				
	Anterior leaflet (%)	—	20 (40)	—
	Posterior leaflet (%)	—	30 (60)	—
Use of annular rings				
	Use	—	50 (100)	—
	No use	—	0 (0)	—
Leaflet resection				
	Resection	—	29 (58)	—
	No resection	—	21 (42)	—
Use of neochordae				
	Use	—	27 (54)	—
	No use	—	23 (46)	—

Values are n, mean ± SD or n (%).

### 3.2 Echocardiographic Parameters in Controls and Patients One Week before and One Month after Mitral Valve Repair

Compared with the control group, the left atrial and LV chamber were enlarged in patients one week before operation with statistically significant differences in LAD (*p *< 0.001), LVEDD (*p* = 0.01), LVEDV (*p *< 0.001), LVESD (*p* = 0.01), and LVESV (*p* = 0.01) (Table 2). Mean transmitral gradient was significantly increased after operation (*p *< 0.001).

**Table 2.** **Comparison of echocardiographic parameters before and after operation**.

Variable	Control group	Mitral Valve Repair group (n = 50)		*p* value
	(n = 30)	Before op 1 week	Post op 1 month	
LAD (mm)	33.45 ± 3.27	42.28 ± 3.14*	34.08 ± ${\mathrm{4.56}}^{†}$	<0.001
LVEDD (mm)	47.14 ± 3.31	55.44 ± 4.90*	47.74 ± ${\mathrm{4.18}}^{†}$	0.01
LVEDV (mL)	103.46 ± 14.26	152.72 ± 29.09*	108.26 ± ${\mathrm{23.15}}^{†}$	<0.001
LVESD (mm)	32.54 ± 2.34	35.98 ± 3.36*	32.84 ± ${\mathrm{3.03*}}^{†}$	0.01
LVESV (mL)	36.39 ± 6.31	56.16 ± 12.36*	43.14 ± ${\mathrm{10.39*}}^{†}$	0.01
LVEF (%)	62.14 ± 2.63	63.00 ± 4.12	60.42 ± 2.19	0.08
EL-AVE(J/s·m)	6.29 ± 1.69	31.64 ± 13.05*	11.33 ± ${\mathrm{3.70*}}^{†}$	<0.001
Mean transmitral gradient	3 (2–4)	—	4 (3–5)*	<0.001

Values are mean ± SD. LAD, left atrial dimension; LVEDD, left ventricular end-diastolic dimension; LVEDV, left ventricular end-diastolic volume; LVESD, left ventricular end-systolic dimension; LVESV, left ventricular end-systolic volume; LVEF, left ventricular ejection fraction; EL-AVE, average energy loss. Note: Compared with control group, * p < 0.05; Compared with 1 week before operation, ^†^* p <* 0.05.

Compared with one week before operation, the left atrial and LV chamber were reduced one month after operation, and the difference in LAD (*p *< 0.001), LVEDD (*p *< 0.001), LVEDV (*p *< 0.001), LVESD (*p* = 0.01), and LVESV (*p* = 0.01) were statistically significant. There was no statistically significant difference in LVEF (*p* = 0.08).

### 3.3 EL-AVE during Diastole in Controls and Patients Who Underwent Mitral Valve Repair

**Fig. 4.**
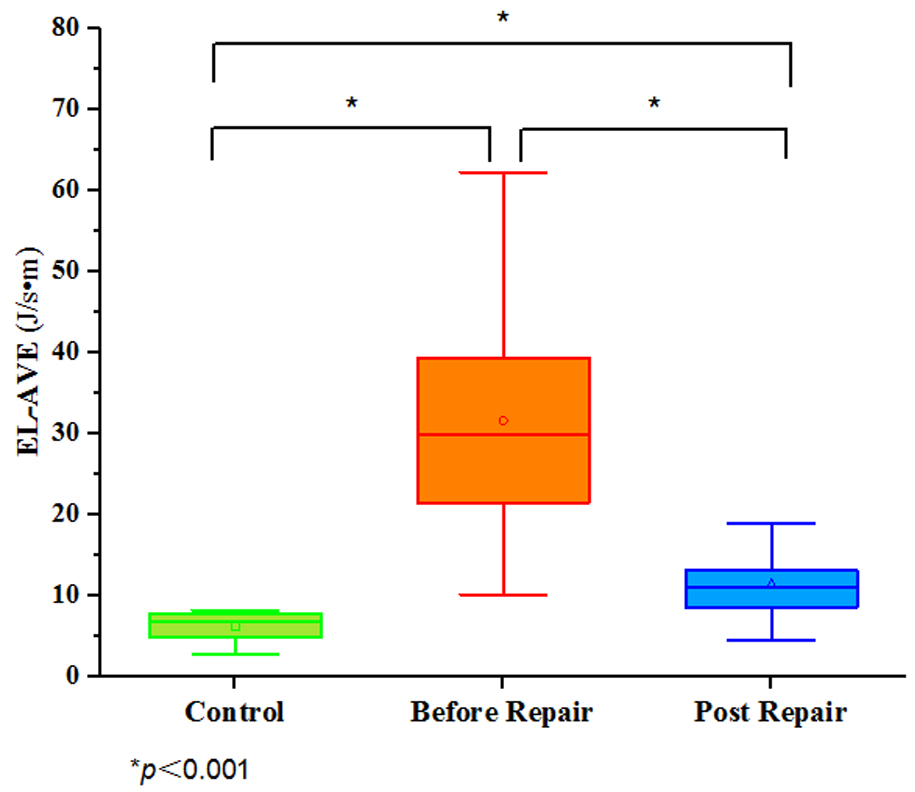
**A statistically significant difference was observed when comparing EL-AVE of the LV during diastole in controls and patients before and after mitral valve repair**. Green: EL-AVE of LV in controls. Orange: EL-AVE of LV in patients who underwent mitral valve repair one week before operation. Blue: EL-AVE of LV in patients who underwent mitral valve repair one month after operation. EL-AVE, average energy loss.

Compared with the control group, the EL-AVE before and after operation in the mitral valve repair group was significantly increased (*p *< 0.001) (Table 2 and Figs. 3,4).

Compared with one week before operation, the EL-AVE after operation in the mitral valve repair group was significantly decreased (*p *< 0.001).

### 3.4 Effect of Surgical Techniques on EL-AVE One Month after Operation

The effect of mitral valve resection on EL-AVE one month after operation was significant (*p *< 0.001), and no significance was observed in the effect of extension of leaflet degeneration and neochordae on EL-AVE one month after operation (*p* = 0.65, 0.20) in Table 3. There was no interaction of these factors on EL-AVE after operation (*p* = 0.98).

**Table 3.** **Effects of surgical technique and extension of leaflet degeneration on EL-AVE**.

		EL-AVE (J/s·m)
Resection		
	with	12.78 ± 3.54
	without	9.33 ± 2.95
*p* value		<0.001
Extension		
	single	10.97 ± 2.88
	two	11.99 ± 4.85
*p* value		0.65
Neochordae		
	with	11.57 ± 3.78
	without	11.05 ± 3.66
*p* value		0.20

Values are mean ± SD. EL-AVE, average energy loss.

### 3.5 Characteristics of EL-AVE in Patients with and without Mitral Leaflet Resection

There were no statistically significant differences between the resection subgroup and the non-resection subgroup, including age (*p* = 0.67), sex distribution (*p* = 0.58), annuloplasty ring size (*p* = 0.39), systolic pressure (*p* = 0.37), diastolic pressure (*p* = 0.06) and BSA (*p* = 0.56).

Differences in the surgical procedure between the two subgroups were as follows. The most common leaflet abnormalities in the resection subgroup were prolapse involving single leaflet, posterior mitral leaflet and no use of neochordae. In the non-resection subgroup, prolapse involving anterior mitral leaflet and use of neochordae were the most common leaflet abnormalities. There was no significant difference in the non-resection subgroup involving single or two leaflet prolapse. Compared with the resection subgroup, the EL-AVE during diastole of the non-resection subgroup was significantly decreased one month after operation (*p *< 0.001) (Table 4).

**Table 4.** **Comparison of characteristics and EL-AVE in patients with and without mitral leaflet resection**.

		Resection subgroup	Non-Resection subgroup	*p* value
		(n = 29)	(n = 21)	
Age, years		56.03 ± 7.44	55 ± 8.05	0.67
Male (%)		16 (55)	13 (62)	0.58
Body Surface Area, $m^{2}$		1.60 ± 0.15	1.63 ± 0.17	0.56
Annuloplasty ring size, mm		30 ± 1.9	30 ± 2.3	0.39
Blood pressure, mm Hg				
Systolic		117.2 ± 9.47	115.6 ± 9.00	0.37
Diastolic		71.1 ± 5.0	69.8 ± 4.0	0.06
Extension of leaflet degeneration				
	Single (%)	22 (76)	10 (47)	<0.001
	Two (%)	7 (24)	11 (53)	<0.001
Location of leaflet degeneration				
	Anterior leaflet (%)	4 (14)	16 (76)	<0.001
	Posterior leaflet (%)	25 (86)	5 (24)	<0.001
Use of neochordae				
	Use	11 (38)	18 (86)	<0.001
	No Use	18 (62)	3 (14)	<0.001
EL-AVE, J/s·m		12.78 ± 3.54	9.33 ± 2.95*	<0.001

Values are n, mean ± SD or n (%). EL-AVE, average energy loss. Note: Compared with resection group, * *p *< 0.05.

### 3.6 Intraobserver and Interobserver Variability

**Fig. 5.**
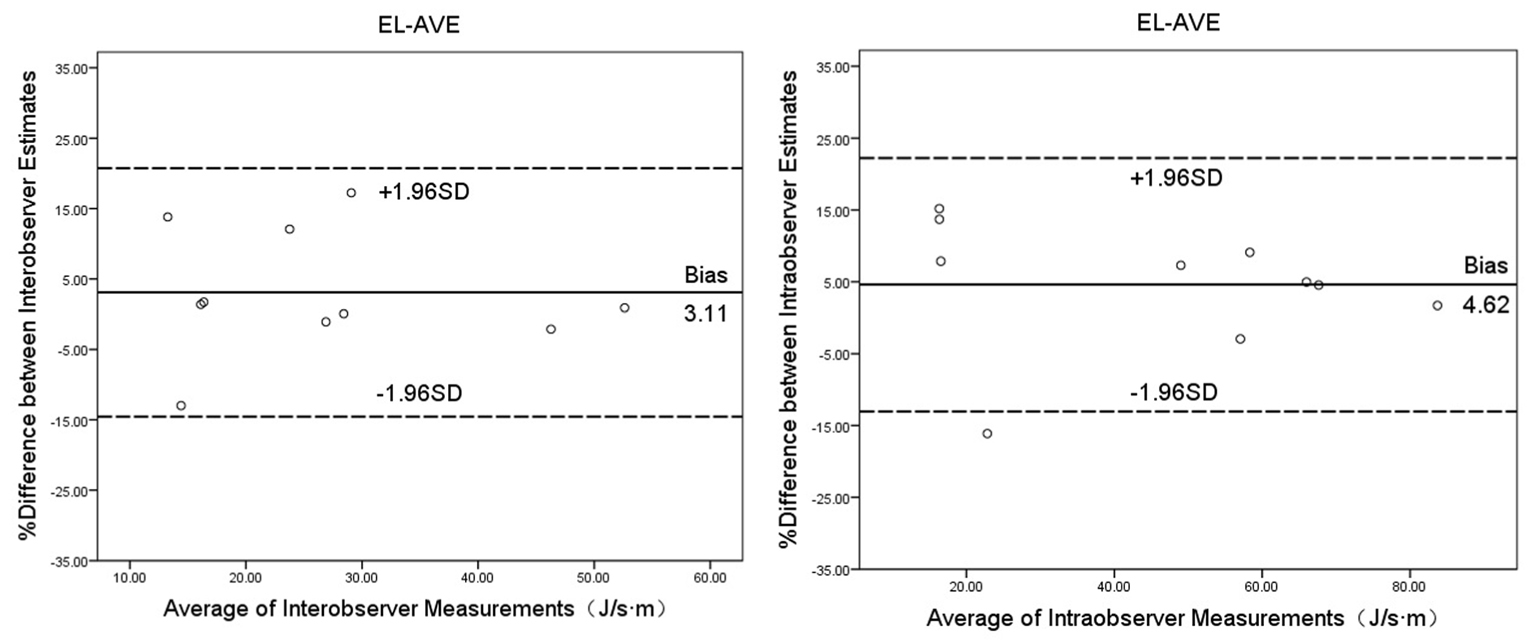
**Bland-Altman plots for interobserver and intraobserver agreement on EL-AVE during diastole**. EL-AVE, energy loss.

The Bland-Altman analysis for assessing the intraobserver (differences 4.62 ± 9.03, 95% CI –13.04~22.24) and interobserver (differences 3.11 ± 9.02, 95% CI –14.54~20.74) variability for EL-AVE one week before operation demonstrated excellent reliability (Fig. 5).

### 3.7 One Month Prognosis after Mitral Valve Repair

Of the fifty patients who underwent mitral valve repair, one patient experienced poor healing of the surgical incision in the resection subgroup, and one patient had hoarseness in the non-resection subgroup, and the rest of the patients recovered well after operation. Postoperative review by echocardiogram showed no significant abnormalities in mitral valve function according to the ASE guidelines [14] with definitions EROA <0.2 ${\mathrm{cm}}^{2}$ and mean pressure gradient <5 mm Hg.

## 4. Discussion

Mitral valve repair has become the preferred surgical procedure for the treatment of patients with severe degenerative mitral regurgitation [16, 17]. The procedure involves partially resection of the posterior mitral leaflet and implantation of a mitral annuloplasty to reshape the annulus and support leaflet repair [18]. The changes in the spatial conformation of the annulus and elevated mitral gradients lead to a change of LV flow pattern and affects the prognosis of patients [19]. Morichi *et al*. [20] reported that energy loss after mitral valve repair was greater than that of healthy volunteers during early diastole, as measured by VFM. This may be due to a different type of annuloplasty ring that was used during mitral valve repair. The relatively small ring resulted in an abnormal LV flow pattern and increase in energy loss.

Our study has two main findings. First, the EL-AVE in patients after mitral valve repair was higher than that of controls, but lower than that before mitral valve repair. Second, mitral valve repair resulted in a higher EL-AVE in patients with resected leaflets than in those with unresected leaflets while the same type of annuloplasty ring was used.

Vortices play an important role in normal cardiac function by keeping blood in motion inside the cardiac chambers and preserving momentum. They create an ideal state of kinetic energy reserve, and accumulation and transport of blood in the early stage of ventricular contraction [21]. The biphasic vortex rings are formed in the early and late LV filling, which is a consequence of the LV chiral asymmetry and the interaction between the blood-filled jet, the wall and the mitral valve [22, 23]. The longer anterior leaflet generates a stronger anterior vortex, while the shorter posterior leaflet generates a weaker posterior vortex. The anterior vortex dominates the posterior vortex, thereby facilitating the transfer of blood and improving the filling efficiency of the LV. The asymmetry of this leaflet creates vortices with preservation of kinetic energy and no energy loss [24].

The energy loss equation shows that it is related to the size and direction of adjacent velocity vectors. Diastolic energy loss refers to the energy lost by shear friction of blood of that flows in the LV after opening of the mitral valve with the ventricular wall [25]. In our study, EL-AVE during mitral regurgitation increased with the severity of mitral regurgitation. This may be due to the change of the size and direction of the intraventricular velocity vectors as a result of the turbulence caused by the mitral regurgitation. EL-AVE increased due to powerful collisions with the ventricular wall.

**Fig. 6.**
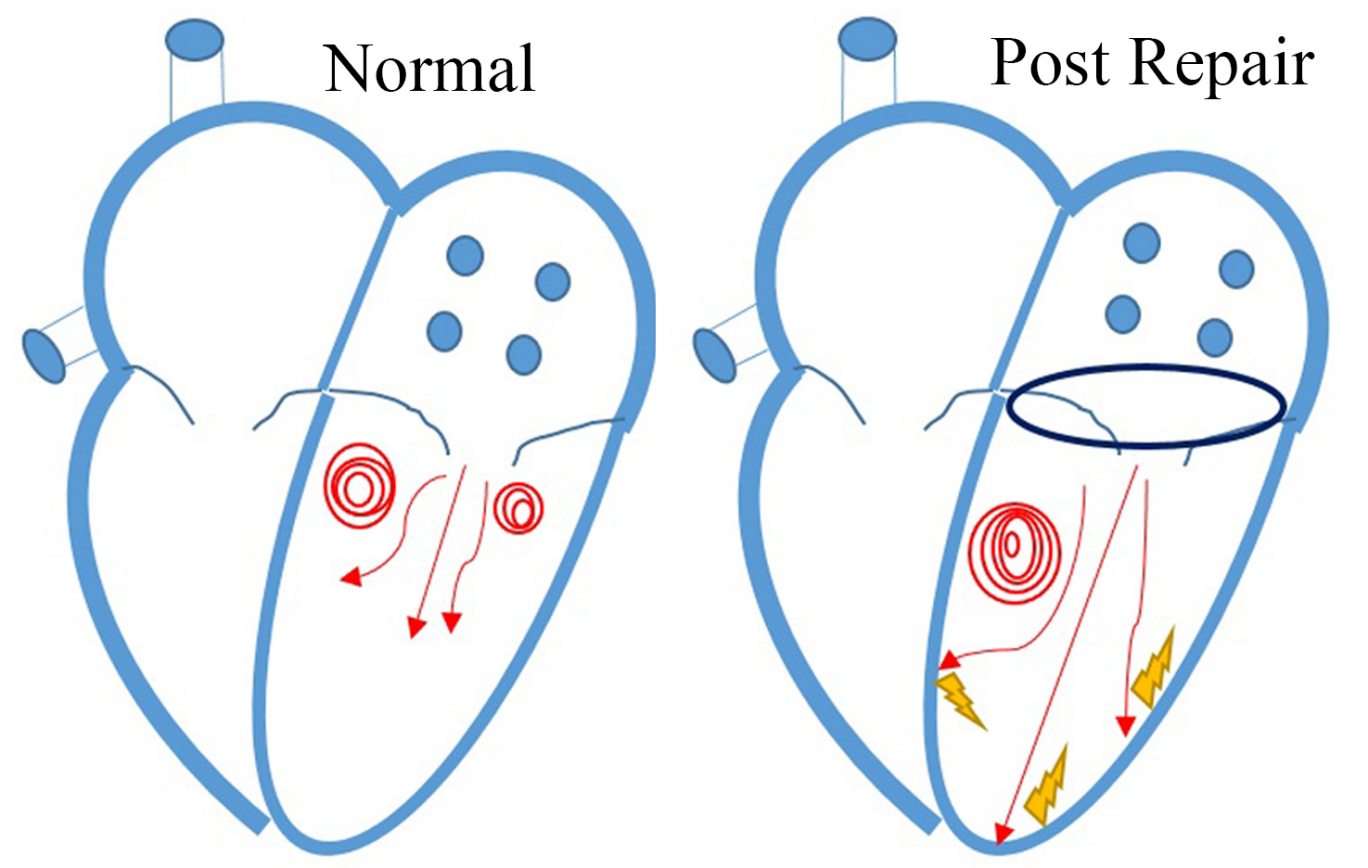
**Vortex change after mitral valve repair during diastole. Normal vortex patterns in a normal LV (left) and after mitral valve repair (right)**. Blood flow dissipated due to the collision of transmitral inflow on the ventricular wall after mitral valve repair.

During diastole, the left intraventricular pressure is reduced by active relaxation of the myocardium and dilatation of the LV. This maximizes the pressure gradient between left atrium and LV, causing withdrawal of blood from the atria and acceleration of blood into the LV. Recent data suggest that functional mitral stenosis may occur following valve repair [26]. Increases in transmitral flow after mitral valve repair leads to turbulent flow above and below the mitral valve, resulting in an increase of energy loss. When the anterior and posterior leaflets of the mitral valve have the same size or the posterior leaflet is short, an increase in energy loss is observed as the blood flow collides on the ventricular wall and the stability of vortices is destroyed (Fig. 6). The aim during mitral valve repair is to preserve the vortex pattern, resulting in a lower energy loss. In our study, the resection subgroup consisted mainly of patients with prolapse of the posterior mitral leaflet. The rigidity of the posterior mitral leaflet after resection restricts the opening of the posterior mitral leaflet, and the transmissive inflow tends to collide on the ventricular wall, resulting in an elevated energy loss. In addition, the transition of the mitral annulus from a saddle D-shape during systole of the cardiac cycle to a flat D-shape during diastole has been confirmed [27]. Compared with the D-shaped mitral annulus morphology, the use of an O-shaped semi-rigid complete ring resulted in more energy loss because of the strength of the dominant vortical structure that was formed and the energy dissipation [28].

The ultimate aim of heart valve surgery is to reduce the cardiac dysfunction by stopping regurgitation or reducing pressure gradients, which are the factors contributing to hemodynamic abnormalities. The superiority of mitral valve repair over replacement for short-term and long-term survival is due to the subvalvular apparatus that is preserved in mitral valve repair. This maintains left ventricular geometry and allows for a reduction in the left ventricular radius. Nevertheless, Chan *et al*. [19] found that elevated mitral gradient correlates with prognosis in patients after mitral valve repair for degenerative mitral valve regurgitation. The intraventricular vortex and intraventricular energy loss are key factors affecting the prognosis after mitral valve surgery [29]. In our study, different surgical techniques resulted in different postoperative EL-AVE. This was especially observed with relatively small effective orifice area that induced abnormal LV flow patterns and increased EL-AVE. A long-term follow-up study is needed to study the effect of EL-AVE increase on cardiac function after mitral valve repair.

## 5. Generalizability

First, mitral valve repair has been widely accepted, due to its superiority over valve replacement regarding long-term survival, fewer valve-related complications, and preservation of the LV function [30]. Second, VFM is safe, effective and non-invasively detects hemodynamic changes in the heart cavity. The reproducibility and generalizability of VFM technology for the evaluation of LV flow patterns in different types of mitral valve surgery have been confirmed in this study [31].

## 6. Limitations

First, the apex of the heart cannot be completely enclosed in patients with significant LV enlargement due to two-dimensional angulation. When there is a defect in the ventricular wall, the EL-AVE may not be accurately measured. Second, when the area of the reflux beam is greater than 50% of the area of the left atrium, the blood flow in the LV cavity may result in aliasing twice, which affects the accuracy of the EL-AVE measurement; Third, the postoperative follow-up time of this study is short, and there is a lack of long-term postoperative sample data. Fourth, the number of samples in this study is small, and further research is necessary to collect more relevant data. Fifth, EL-AVE is only applicable to patients in sinus rhythm in this study. Whether it is applicable to all patients regardless of rhythm will require further studies to determine.

## 7. Conclusions

In summary, the LV flow patterns of patients with mitral valve repair can be quantitatively evaluated. Moreover, a greater energy loss was observed in patients after mitral valve repair than in healthy volunteers. The mitral leaflet resection and complete rings changed LV flow patterns, resulting in changed energy loss distribution. Different surgical techniques can affect the changes of energy loss after operation, especially in patients with a relatively small effective orifice area. A potential role for VFM in clinical decision-making merits further investigation.
